# Effects of Immediate Oxygen Supplementation (Sodium Carbonate and Hydrogen Peroxide) on Water Quality Parameters, Behavioural Responses and Survival of *Puntius sophore* Fingerlings

**DOI:** 10.1002/vms3.70624

**Published:** 2025-09-26

**Authors:** Shishir Kumar Nandi, Arman Hossain, Shamima Nasren, Muhammad Anamul Kabir, Md. Abdullah Al Mamun

**Affiliations:** ^1^ Department of Aquaculture Faculty of Fisheries Sylhet Agricultural University Sylhet Bangladesh; ^2^ Laboratory of Fish Diseases Diagnosis and Pharmacology Department of Fish Health Management Faculty of Fisheries Sylhet Agricultural University Sylhet Bangladesh; ^3^ Department of Fish Biology and Genetics Faculty of Fisheries Sylhet Agricultural University Sylhet Bangladesh

**Keywords:** aquaculture management, bio‐ox, immediate oxygenation, Kaplan–Meier survival, *Puntius sophore*, water quality parameters

## Abstract

The intensification of aqua‐farming practices has increased the need for a constant oxygen supply to avert oxygen depletion and unexpected fish mortality in captivity. This study assessed the effects of bio‐ox (10% sodium carbonate and hydrogen peroxide) as an immediate oxygen booster on water quality, behavioural patterns and cumulative per cent survival (CPS) of *Puntius sophore* (3.50 ± 0.13 g) in a controlled rearing environment. The completely randomized design comprised four treatments: a control group (T1) without bio‐ox and three experimental groups (T2, T3 and T4) treated with 1, 3 and 5 g of bio‐ox per 70 L of water, respectively. Bio‐ox was applied at the start and at 36 h, with observations recorded every 6 h. Initially, water quality parameters showed no significant differences (*p* > 0.05) among treatments. Post‐supplementation, bio‐ox levels significantly influenced (*p *< 0.05) hydrological variables, including conductivity, total dissolved solid (TDS), total ammonia nitrogen (TAN), dissolved oxygen (DO) and pH. Conductivity and TDS increased with higher bio‐ox dosages, whereas TAN levels were significantly lower (*p* < 0.05) in all bio‐ox–treated groups compared to the control, with no significant differences among treated groups at 42, 48 and 54 h. DO peaked at 11.90 ± 0.10 mg/L at 60 h in T4, whereas T2 maintained optimal levels. The pH also varied significantly (*p* < 0.05), reaching a maximum of 9.23 ± 0.01 at 36 h in T4. Temperature, water pressure and salinity remained unaffected (*p* > 0.05). Behavioural observations indicated minimal abnormalities in T2, whereas higher bio‐ox doses (T3 and T4) resulted in pronounced changes, including hyperactivity, abnormal swimming and convulsions. Kaplan–Meier survival analysis revealed significant differences among treatments, with T2 showed the highest CPS of 66.7%, which was significantly greater than that of T3 (26.7%, *p* = 0.026) and T4 (13.3%, *p* = 0.007). The control group T1 (40%) exhibited intermediate survival rates, which were not significantly different from any treatment groups. These findings suggest that bio‐ox at 1 g/70 L (T2) provides optimal conditions, stabilizing fish behaviour and significantly improving survival compared to higher doses.

## Graphical abstract

•The graphical abstract shows that bio‐ox at 1 g/70 L effectively increases dissolved oxygen, maintains calm fish behaviour and improves survival rates.

•Higher oxygen‐supplementing dosages further increase pH and DO but trigger stress responses such as hyperactivity and convulsions, reducing survival.

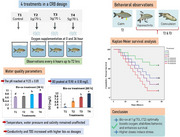

## 1 Introduction

The aquaculture industry has emerged as a highly promising sector, providing a sustainable solution to meet the rising global demand for fish and seafood while safeguarding the long‐term viability of aquatic resources (Mukhtar et al. 2025). In 2020, the worldwide aquaculture production achieved an unprecedented milestone, surpassing all previous records, with an impressive output of 122.6 Mt and a total worth of $281.5 billion (FAO 2022). This remarkable growth underscores the importance of aquaculture in global food security and economic growth (Ababouch et al. 2023). To meet the demand for higher yields, the aquaculture sector has seen a significant shift from extensive to intensive culture systems (Rocha et al. 2022). These include tank culture, biofloc technology (BFT), recirculating aquaculture systems (RAS) and flow‐through systems (Ahmed and Turchini 2021). All of these are designed to maximize production while minimizing environmental impact (McCusker et al. 2023; Zhao et al. 2023; Abdul‐Qadir et al. 2024; Minaz et al. 2024). Among these methods, BFT has gained popularity as an environmentally friendly and efficient system that promotes sustainable fish production. This high‐density stocking increases productivity, making biofloc especially valuable in intensive systems, where maximizing yield and minimising resource use are critical goals (Ogello et al. 2021). However, intensive systems face the challenge of maintaining stable oxygen levels, a vital factor for fish health (Hridoy et al. 2025).

Oxygen is crucial for the metabolic processes of fish (Rubalcaba et al. 2020), and insufficient oxygen can lead to stress, stunted growth and even mortality (Chowdhury and Saikia 2020; Bulbul Ali and Mishra 2022). In developing countries, intensive aquaculture system is rapidly expanding. Frequent power outages create additional challenges by disrupting aeration systems that rely on continuous electricity. These interruptions can cause rapid declines in dissolved oxygen (DO) levels, threatening fish survival and highlighting the urgent need for alternative oxygen sources during power outages or aeration failures (Mwesigwa 2008). In Bangladesh, biofloc systems have gained popularity for small‐scale, backyard setups and are particularly appealing to unemployed youth, offering a profitable, low‐cost farming model (Achariya et al. 2025). However, this modern aquaculture approach faces challenges due to frequent power disruptions in Bangladesh. The country is currently grappling with energy shortages and importing power from India (Sarker et al. 2024). Frequent power disruptions in Bangladesh, often lasting 2–3 days, complicate the use of aeration‐dependent systems, leaving farmers reliant on alternative solutions to maintain oxygen levels and ensure stock survival. The oxygen supplier provides a practical option, delivering stable oxygenation to support fish health during extended blackouts. This is especially important in biofloc setups where zero water exchange can lead to poor water quality and oxygen depletion (Zhang et al. 2023). Even in traditional pond aquaculture systems, oxygen levels dropped significantly during the monsoon season. This occurred when continuous cloudy skies and prolonged rainfall reduced natural oxygen replenishment (Pillay 2008). This decline in oxygen levels posed a serious risk to fish health, particularly for Indian major carps (IMCs). IMCs are dominant in the aquaculture industry and highly sensitive to oxygen fluctuations. These conditions created an urgent need for reliable oxygen‐supplementing solutions to prevent fish stress and mortality (Azmat et al. 2016; López‐Jiménez et al. 2024).

In Bangladesh, over 20 companies have marketed oxygen‐supplying agents for aquaculture use, highlighting the demand for such products (Paul et al. 2021). These products primarily contained active ingredients such as oxidizing agents, hydrogen peroxide and sodium carbonate, which released oxygen upon application, thereby enhancing water quality and supporting fish health (Rahman et al. 2017) both in modern and traditional aquaculture practices. Bio‐ox, marketed by ACI Animal Health, a division of ACI Limited, is a widely used oxygen‐supplying agent in aquaculture (Paul et al. 2021). Bio‐ox is composed of sodium carbonate and 10% hydrogen peroxide. It provides reliable oxygen stabilization and helps safeguard aquaculture stocks during periods of low DO, ensuring healthier aquatic environments. Both in traditional pond and intensive tank aquaculture, barbs (*Puntius* spp.) and tilapia species are commonly farmed due to their adaptability and market demand (Sarker et al. 2002; Rathore et al. 2021).

In the current study, *Puntius sophore* was chosen as a model organism because of its reliance on DO. Unlike air‐breathing species such as catfish, *P. sophore* depends solely on waterborne oxygen, making it a suitable model for evaluating the efficacy of bio‐ox as an oxygen‐supplementing solution. The spot‐fin swamp barb (*P. sophore*), a member of the Cyprinidae family, is widely distributed in rivers, streams and ponds throughout Bangladesh, Bhutan, India, Nepal, Pakistan, Afghanistan, China and Myanmar (Rana et al. 2020). This species holds high nutritional value, containing approximately 18.53% crude protein, 2.28% crude lipid and 3.56% crude ash on a wet basis (Ahmed et al. 2012), making it an attractive candidate for aquaculture. However, *P. sophore* has become scarce and highly valued due to factors such as inadequate water quality management, habitat degradation, overexploitation, pollution, siltation, disease outbreaks and the introduction of alien species (Toufique et al. 2014; Ali et al. 2016). To the best of our knowledge, this is the first study to demonstrate the effects of varying dosages of the oxygen supplements (bio‐ox) on water quality, behavioural responses and survival patterns of *P. sophore* in a controlled environment. This research provides new insights into the potential of this oxygen supplier as an alternative solution for oxygen supplementation, particularly in scenarios where stable oxygen levels are critical, such as during power outages or low‐oxygen conditions in aquaculture environments.

## 2 Materials and Methods

### 2.1 Study Location

The present research was conducted at Wet Laboratory of Fish Health Management Department, Sylhet Agricultural University, Bangladesh in September, 2023.

### 2.2 Fish Collection and Acclimatization

The fingerlings (*n* = 200) of *P. sophore* (3.50 ± 0.13 g) were collected from the Reliance Aqua Farms, Trishal, Mymensingh, Bangladesh. Upon collection, the fish were transported to the laboratory facility in oxygenated airtight plastic bags to maintain optimal oxygen levels. Subsequently, the fingerlings were acclimatized with the laboratory conditions for 7 days in a rectangular glass aquarium (1000 L). During the acclimatization period, the fish were given a commercial floating feed (containing 35% crude protein, 6% crude lipid and 16% crude ash) once daily. At that time, the water in the glass tank was maintained at a temperature of 26.6–26.8°C, DO of 5.40–6.03 mg/L, pH of 6.8–7.2 and total ammonia of 0–0.25 mg/L. At the end of acclimatization, about 120 healthy and energetic fish were chosen and distributed randomly into 12 tanks (70 L) with a stocking of 15 fish in each tank (73 × 35 × 38 cm^3^).

### 2.3 Experimental Design and Trial

The bio‐ox (containing 10% sodium carbonate and H_2_O_2_ as active ingredients) was procured from ACI Animal Health Division, ACI Pvt. Ltd., Bangladesh. This study used a completely randomized design with four treatments, including control (without bio‐ox, T1) and three experimental groups with varying levels of bio‐ox supplementation: 1 (T2), 3 (T3) and 5 g (T4) for 70 L of water in each tank. Each treatment was carried out in triplicate. Bio‐ox was administered at the beginning of the experiment and again at 36 h, with water quality effects monitored at 6‐h intervals over a 72‐h period.

### 2.4 Measurement of Water Quality Parameters

The hydrological variables such as temperature (°C), water pressure (mm, Hg), conductivity (Siemens/m), total dissolved solid (TDS) (mg/L), DO (mg/L), salinity (ppt) and pH of each bio‐ox supplemented tank water were analysed using a multiparameter probe (HI 9828, YSI Incorporation, Yellow Spring, OH, USA). This study involved measuring the parameters at different time intervals after applying bio‐ox into water (0, 6, 12, 18, 24, 30, 36, 42, 48, 54, 60, 66 and 72 h). In brief, the multiparameter probe was subjected to calibrate prior to each sampling event following the manufacturer specifications. The probe was outfitted with sensors, allowing simultaneous measurement of multiple parameters. Subsequently, the probe was submerged into water at a standardized depth, and the measurement of each parameter was recorded. In the meantime, the ammonia (total ammonia nitrogen [TAN]) content of water was detected employing a HACH test kit (HI 28049, HACH, USA).

### 2.5 Behavioural Assessment of *P. sophore* Exposed to Bio‐Ox Supplementation

The research examined the behavioural anomalies in experimental fish using the approach outlined in the study by Akter et al. (2024). Observations were conducted daily in the fish holding tanks for both the control group and the bio‐ox‐treated groups to detect any behavioural alterations. The study noted behavioural changes categorized as follows: no abnormalities (−), slight abnormalities (+), moderate abnormalities (++), above moderate abnormalities (+++) and severe abnormalities (++++).

### 2.6 Survival and Statistical Analysis

The survival of *P. sophore* was monitored at 6‐h intervals over a 72 h experimental period under varying concentrations of bio‐ox. According to Rahman et al. (2025), the Kaplan–Meier method was used to estimate cumulative per cent survival (CPS), with mortality events recorded as 1 and fish surviving beyond the observation period treated as censored cases (coded as 0). Kaplan–Meier survival curves were plotted, and censored data were indicated by “+” symbols. Statistical comparisons of survival distributions were conducted using the log‐rank (Mantel–Cox) test for both overall and pairwise comparisons among treatment groups. Additionally, all experimental data, including water quality parameters, were analysed using SPSS version 26.0. One‐way ANOVA followed by Duncan's multiple range test was applied to identify significant differences among treatments. All results were expressed as mean ± standard deviation (SD), and a threshold of *p* < 0.05 was used to determine statistical significance throughout the study.

## 3 Results

### 3.1 Analysis of Tank Water Hydrological Parameters

**FIGURE 1:**
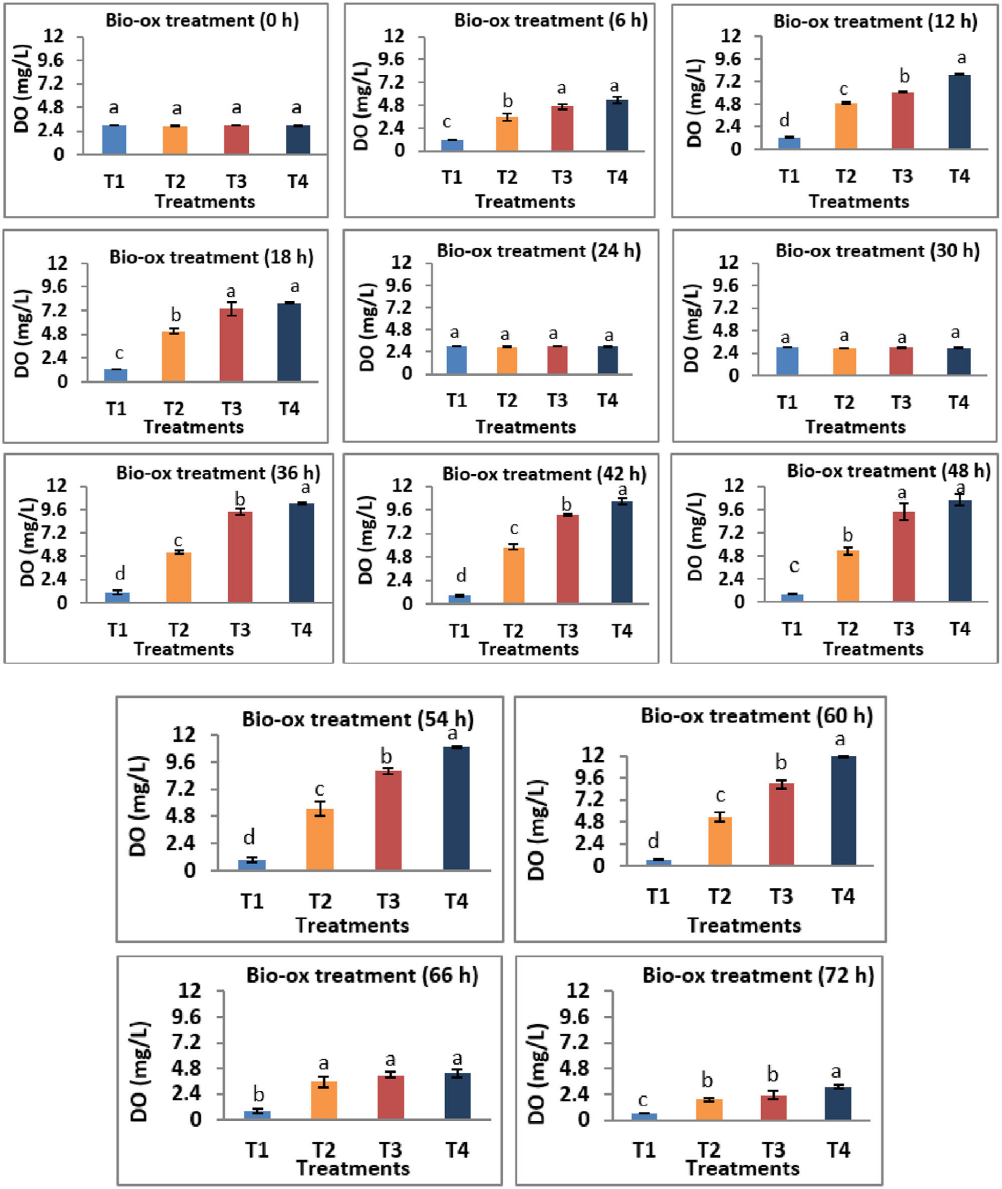
Effects of different supplementation levels of bio‐ox on dissolved oxygen content of tank water. The data are presented as mean ± SD, and bars with different superscripts indicate significant differences (*p* < 0.05).

**FIGURE 2:**
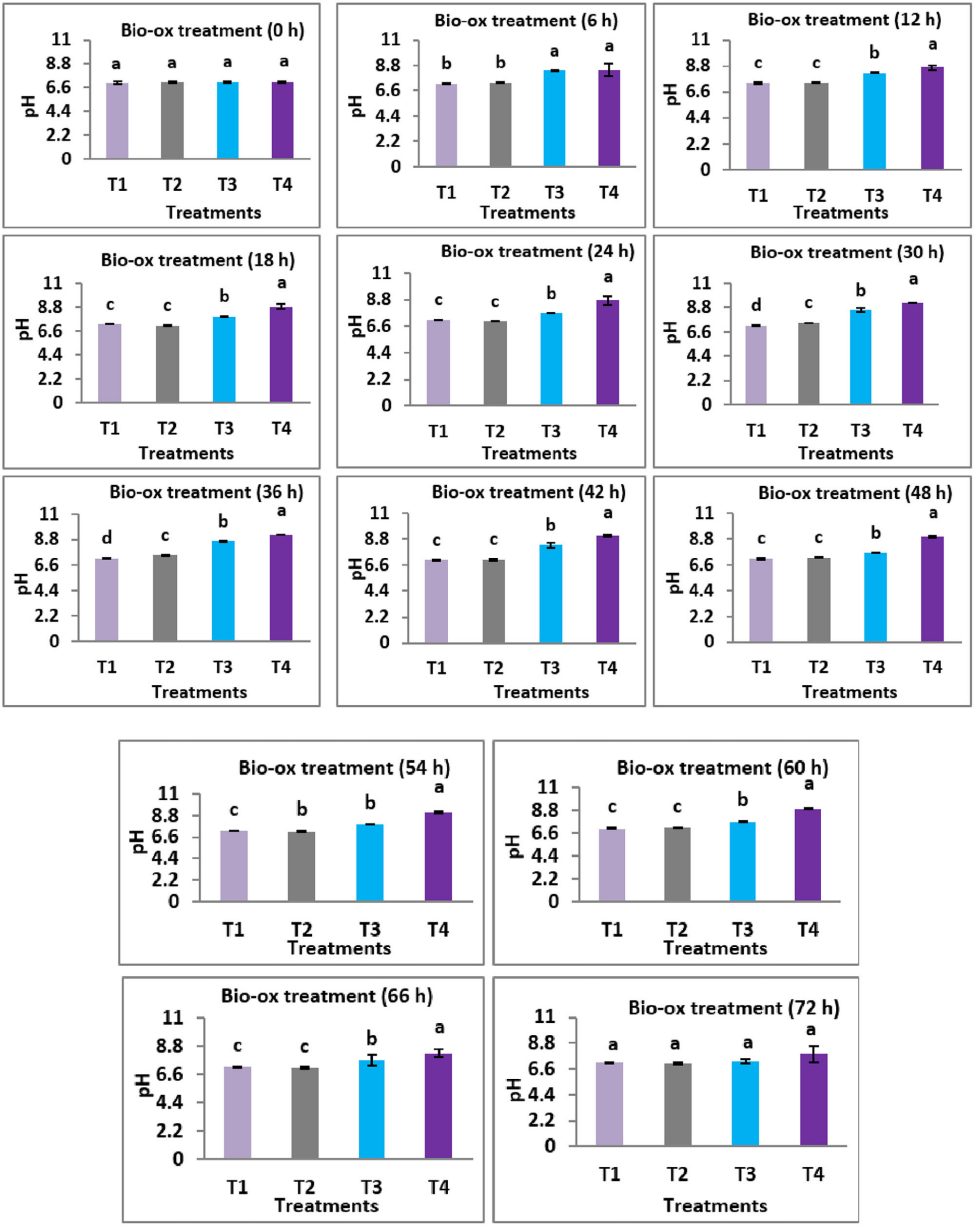
Effects of different supplementation levels of bio‐ox on pH content of tank water. The data are presented as mean ± SD, and bars with different superscripts indicate significant differences (*p* < 0.05).

The water quality parameters of fish holding tank water after being treated with various levels of bio‐ox supplementation are presented in Table 1 and illustrated in Figures 1 and 2. At the onset of the experiment (0 h), all the water variables, such as temperature, water pressure, conductivity, TDS, salinity, total ammonia, DO and pH, remained insignificant (*p* > 0.05) among the treatment groups. Following the bio‐ox administration, the results of this observation revealed that different degrees of bio‐ox had notable (*p *< 0.05) impacts on the majority of the water quality variables such as conductivity, TDS, total ammonia, DO and pH contents. However, the mean values of temperature, water pressure and salinity did not show significant variations (*p* > 0.05) when the experimental tank water underwent various concentrations of bio‐ox, with the exception of the temperature level after the 12‐h bio‐ox treatment. Interestingly, there was a prominent rise (*p* < 0.05) in the conductivity and TDS levels with the increase of supplementation levels of bio‐ox in the water. In contrast, the control group exhibited the significantly (*p *< 0.05) down levels of conductivity and TDS. The application of bio‐ox demonstrated a significant influence on total ammonia content. As the dosage of bio‐ox increased in this study, there was a noticeable decrease in total ammonia levels. Furthermore, the variations in treatment groups did not have a considerable impact (*p* > 0.05) on total ammonia levels during the 42, 48 and 54 h of observation. However, the treatment without bio‐ox showed significantly higher (*p* < 0.05) values of total ammonia.

**TABLE 1:** Hydrological variables of tank water after being treated with different levels of bio‐ox supplementation.

Observations time points (h)	Parameters	Treatments (bio‐ox, gram)			
		T1 (0)	T2 (1)	T3 (3)	T4 (5)
0 h	Temperature (°C)	26.45 ± 0.07	26.45 ± 0.21	26.50 ± 0.00	26.40 ± 0.14
	W. pressure (mm, Hg)	758.05 ± 0.07	758.03 ± 0.08	758.05 ± 0.09	758.05 ± 0.06
	Conduct. (Siemens/m)	137.00 ± 1.00	136.74 ± 0.38	135.96 ± 0.06	136.40 ± 1.03
	TDS (mg/L)	70.33 ± 0.58	71.00 ± 1.00	70.63 ± 0.55	70.98 ± 0.03
	Salinity (ppt)	0.07 ± 0.01	0.08 ± 0.01	0.08 ± 0.01	0.08 ± 0.01
	TAN (mg/L)	0.63 ± 0.01	0.62 ± 0.01	0.64 ± 0.02	0.62 ± 0.01
6 h	Temperature (°C)	26.05 ± 0.07	26.05 ± 0.08	26.06 ± 0.09	26.05 ± 0.08
	W. pressure (mm, Hg)	753.46 ± 0.35	753.55 ± 0.36	753.65 ± 0.07	753.46 ± 0.26
	Conduct. (Siemens/m)	145.85 ± 1.20^d^	165.60 ± 0.85^c^	228.05 ± 1.34^b^	276.00 ± 5.66^a^
	TDS (mg/L)	72.75 ± 0.35^d^	81.50 ± 0.71^c^	107.75 ± 1.06^b^	130.89 ± 0.91^a^
	Salinity (ppt)	0.08 ± 0.01	0.08 ± 0.02	0.08 ± 0.00	0.08 ± 0.01
	TAN (mg/L)	3.00 ± 0.71^a^	1.25 ± 0.00^b^	0.50 ± 0.00^b^	1.53 ± 1.01^b^
12 h	Temperature (°C)	26.2 ± 0.00^b^	26.50 ± 0.00^a^	26.35 ± 0.07^ab^	26.40 ± 0.14^ab^
	W. pressure (mm, Hg)	753.60 ± 0.09	753.60 ± 0.10	753.60 ± 0.11	753.69 ± 0.08
	Conduct. (Siemens/m)	152.45 ± 0.78^d^	173.16 ± 0.35^c^	231.06 ± 1.33^b^	277.21 ± 8.12^a^
	TDS (mg/L)	74.12 ± 0.12^c^	82.21 ± 0.19^c^	111.00 ± 0.11^b^	135.91 ± 0.99^a^
	Salinity (ppt)	0.07 ± 0.02	0.07 ± 0.01	0.07 ± 0.01	0.08 ± 0.02
	TAN (mg/L)	1.75 ± 0.00^a^	1.50 ± 0.35^a^	1.00 ± 0.00^b^	0.75 ± 0.00^b^
18 h	Temperature (°C)	26.40 ± 0.00	26.40 ± 0.01	26.40 ± 0.02	26.40 ± 0.01
	W. pressure (mm, Hg)	753.60 ± 0.10	753.60 ± 0.11	753.61 ± 0.13	753.61 ± 0.14
	Conduct. (Siemens/m)	155.5 ± 6.36^c^	170.65 ± 0.92^c^	226.00 ± 8.49^b^	273.30 ± 18.81^a^
	TDS (mg/L)	73.55 ± 0.71^d^	84.00 ± 1.41^c^	112.00 ± 1.93^b^	136.50 ± 3.54^a^
	Salinity (ppt)	0.07 ± 0.01	0.08 ± 0.02	0.08 ± 0.02	0.08 ± 0.01
	TAN (mg/L)	1.88 ± 0.18^a^	1.00 ± 0.00^b^	1.00 ± 0.00^b^	0.88 ± 0.18^b^
24 h	Temperature (°C)	26.55 ± 0.07	26.60 ± 0.00	26.50 ± 0.00	26.60 ± 0.14
	W. pressure (mm, Hg)	756.6 ± 0.19	756.3 ± 0.20	756.8 ± 0.21	756.6 ± 0.20
	Conduct. (Siemens/m)	153.80 ± 1.13^d^	171.00 ± 1.41^c^	232.75 ± 3.89^b^	283.10 ± 4.09^a^
	TDS (mg/L)	78.50 ± 4.95^c^	82.03 ± 1.41^c^	113.75 ± 1.06^b^	140.75 ± 0.09^a^
	Salinity (ppt)	0.07 ± 0.00	0.07 ± 0.01	0.07 ± 0.00	0.08 ± 0.01
	TAN (mg/L)	1.50 ± 0.00^a^	1.00 ± 0.00^b^	1.50 ± 0.00^a^	1.13 ± 0.18^b^
30 h	Temperature (°C)	26.55 ± 0.07	26.65 ± 0.07	26.60 ± 0.00	26.60 ± 0.00
	W. pressure (mm, Hg)	755.90 ± 0.1	755.80 ± 0.1	755.90 ± 0.3	755.87 ± 0.13
	Conduct. (Siemens/m)	141.65 ± 2.33^d^	210.95 ± 1.34^b^	319.15 ± 1.20^a^	189.98 ± 14.11^c^
	TDS (mg/L)	77.26 ± 3.89^d^	102.50±0.72^c^	153.50 ± 2.12^b^	203.21 ± 11.67^a^
	Salinity (ppt)	0.08 ± 0.01	0.09 ± 0.01	0.08 ± 0.00	0.08 ± 0.00
	TAN (mg/L)	1.38 ± 0.18^a^	1.25 ± 0.00^a^	0.88 ± 0.18^b^	0.75 ± 0.00^b^
36 h	Temperature (°C)	26.80 ± 0.00	26.80 ± 0.01	26.80 ± 0.00	26.79 ± 0.04
	W. pressure (mm, Hg)	752.80 ± 0.00	752.81 ± 0.01	752.80 ± 0.02	752.82 ± 0.02
	Conduct. (Siemens/m)	157.68 ± 1.58^d^	209.11 ± 0.15^c^	322.50 ± 2.12^b^	431.50 ± 0.71^a^
	TDS (mg/L)	78.50 ± 2.12^d^	100.75 ± 1.06^c^	154.77 ± 3.16^b^	209.55 ± 0.78^a^
	Salinity (ppt)	0.09 ± 0.01	0.09 ± 0.02	0.08 ± 0.01	0.08 ± 0.00
	TAN (mg/L)	1.88 ± 0.18^a^	1.88 ± 0.18^a^	1.75 ± 0.00^a^	1.25 ± 0.00^b^
42 h	Temperature (°C)	27.05 ± 0.07	26.85 ± 0.07	26.85 ± 0.07	26.80 ± 0.14
	W. pressure (mm, Hg)	754.20 ± 0.29	754.21 ± 0.28	754.21 ± 0.19	754.19 ± 0.29
	Conduct. (Siemens/m)	175.15 ± 6.86^c^	202.65 ± 0.49^b^	323.85 ± 5.44^a^	206.75 ± 3.18^b^
	TDS (mg/L)	82.25 ± 1.06^d^	97.75 ± 0.35^c^	155.75 ± 1.06^b^	202.20 ± 1.41^a^
	Salinity (ppt)	0.08 ± 0.01	0.07 ± 0.00	0.08 ± 0.01	0.07 ± 0.00
	TAN (mg/L)	1.25 ± 0.00	1.25 ± 0.00	1.00 ± 0.00	1.00 ± 0.00
48 h	Temperature (°C)	26.95 ± 0.07	26.95 ± 0.07	27.00 ± 0.00	27.00 ± 0.00
	W. pressure (mm, Hg)	753.20 ± 0.12	753.20 ± 0.12	753.21 ± 0.13	753.21 ± 0.14
	Conduct. (Siemens/m)	171.95 ± 11.38^d^	214.75 ± 6.72^c^	333.90 ± 5.52^b^	415.06 ± 22.70^a^
	TDS (mg/L)	79.50 ± 0.71^d^	101.55 ± 2.19^c^	161.35 ± 1.91^b^	214.00 ± 9.90^a^
	Salinity (ppt)	0.06 ± 0.01	0.06 ± 0.02	0.07 ± 0.02	0.06 ± 0.00
	TAN (mg/L)	1.00 ± 0.00	1.00 ± 0.00	0.88 ± 0.18	0.75 ± 0.00
54 h	Temperature (°C)	26.65 ± 0.07	26.70 ± 0.14	26.55 ± 0.07	26.70 ± 0.28
	W. pressure (mm, Hg)	756.50 ± 0.12	756.51 ± 0.13	756.50 ± 0.09	756.52 ± 0.10
	Conduct. (Siemens/m)	148.40 ± 0.85^b^	156.85 ± 11.10^b^	223.00 ± 2.83^a^	237.00 ± 16.97^a^
	TDS (mg/L)	74.50 ± 0.71^d^	82.00 ± 1.41^c^	108.86 ± 0.91^b^	131.36 ± 1.62^a^
	Salinity (ppt)	0.09 ± 0.01	0.08 ± 0.01	0.09 ± 0.01	0.09 ± 0.01
	TAN (mg/L)	1.13 ± 0.18	0.75 ± 0.00	0.88 ± 0.53	0.88 ± 0.18
60 h	Temperature (°C)	25.90 ± 0.00	25.90 ± 0.00	25.85 ± 0.07	26.20 ± 0.57
	W. pressure (mm, Hg)	755.60 ± 0.61	755.63 ± 0.62	755.63 ± 0.63	755.65 ± 0.60
	Conduct. (Siemens/m)	148.35 ± 0.92^c^	159.60 ± 0.00^c^	223.95 ± 5.59^b^	267.10 ± 10.04^a^
	TDS (mg/L)	72.50 ± 3.54^c^	82.25 ± 1.77^c^	110.50 ± 0.71^b^	131.00 ± 1.41^a^
	Salinity (ppt)	0.06 ± 0.00	0.06 ± 0.00	0.06 ± 0.00	0.07 ± 0.01
	TAN (mg/L)	0.88 ± 0.18^a^	0.63 ± 0.18^ab^	0.63 ± 0.18^ab^	0.25 ± 0.00^b^
66 h	Temperature (°C)	26.35 ± 0.35	26.35 ± 0.35	26.45 ± 0.21	26.35 ± 0.49
	W. pressure (mm, Hg)	753.10 ± 0.09	753.02 ± 0.05	753.02 ± 0.01	753.01 ± 0.01
	Conduct. (Siemens/m)	153.50 ± 9.19^d^	171.65 ± 4.74^c^	229.20 ± 1.13^b^	284.75 ± 0.35^a^
	TDS (mg/L)	73.50 ± 2.12^c^	82.25 ± 1.06^c^	115.00 ± 5.66^b^	141.50 ± 4.50^a^
	Salinity (ppt)	0.09 ± 0.01	0.09 ± 0.02	0.08 ± 0.00	0.08 ± 0.01
	TAN (mg/L)	1.88 ± 0.18^a^	1.00 ± 0.00^b^	1.00 ± 0.00^b^	0.25 ± 0.00^c^
72 h	Temperature (°C)	25.90 ± 0.00	25.90 ± 0.00	25.95 ± 0.07	25.95 ± 0.07
	W. pressure (mm, Hg)	755.60 ± 0.60	755.60 ± 0.61	755.63 ± 0.60	755.62 ± 0.60
	Conduct. (Siemens/m)	153.75 ± 8.84^d^	170.15 ± 2.62^c^	229.45 ± 0.78^b^	284.75 ± 0.35^a^
	TDS (mg/L)	74.50 ± 0.71^c^	75.50 ± 2.12^c^	110.50 ± 0.71^b^	146.50 ± 3.54^a^
	Salinity (ppt)	0.09 ± 0.02	0.08 ± 0.01	0.09 ± 0.02	0.08 ± 0.00
	TAN (mg/L)	0.88 ± 0.18^a^	0.75 ± 0.00^ab^	0.50 ± 0.35^ab^	0.25 ± 0.00^b^

*Note*: The data are presented as mean ± SD, with different superscripts indicating statistically significant differences among treatments (*p* < 0.05). The data are presented as mean ± standard deviation (SD).

Abbreviations: Conduct., conductivity; TAN, total ammonia nitrogen; TDS, total dissolved solid; W. pressure, water pressure.

After the bio‐ox treatment, DO levels were highly fluctuated among the various treatment groups in this study. The DO content was significantly (*p *< 0.05) raised with the increase of bio‐ox dosages, and the highest value was noted in T4 treatment throughout the observation period. The maximum DO level was 11.90 ± 0.10 mg/L in T4 group at 60 h of treatment. However, the DO concentration recorded in T2 treatment was in optimum levels for fish's survival over 72 h of experiment. On the other hand, a significantly lower (*p* < 0.05) DO was observed in T1 group.

Furthermore, the water pH demonstrated an ascending pattern, reaching significantly higher values (*p* < 0.05) at 30 and 36 h (9.23 ± 0.01) in the T4 treatment compared to other treatments in the current study. However, the fish groups subjected to T1 and T2 treatments exhibited pH levels suitable for fish production.

### 3.2 Behavioural Observation of Experimental Fish

Table 2 outlines the variations in behaviour exhibited by *P. sophore* upon exposure to varying concentrations of bio‐ox supplementation in the tank water. The findings indicated that the behavioural changes in fish, including hyperactivity, abnormal swimming, loss of equilibrium, mucus secretion, operculum movement, reduced feeding rate, depigmentation and convulsions, were largely influenced by different concentrations of bio‐ox. The most pronounced abnormal behaviours were observed in the T4 treatment. Nonetheless, the test fish exhibited normal behaviour under T2 treatment when compared to other groups that received bio‐ox treatments.

**TABLE 2:** Effects of various levels of bio‐ox administration on behavioural responses of experimental fish during the trial period.

	Treatments (bio‐ox, gram)			
Parameters	T1 (0)	T2 (1)	T3 (3)	T4 (5)
Hyperactivity	−	−	−	+
Abnormal swimming	+++	+	+++	++++
Loss of equilibrium	+	−	++	++
Mucus secretion	−	−	+	+
Operculum movement ++	−	++	+++	
Feeding rate reduction ++	+	++	+++	
Depigmentation	−	−	−	+
Convulsions	−	−	−	+

*Note*: (−): no behavioural changes, (+): slight, (++): moderate, (+++): above moderate and (++++): severe abnormalities.

### 3.3 CPS of *P. sophore*

The Kaplan–Meier survival analysis showed distinct differences in cumulative survival probabilities among the four treatment groups (T1, T2, T3 and T4) over the 72‐h observation period. The log‐rank (Mantel–Cox) test revealed statistically significant difference among the groups (chi‐square = 9.439, df = 3, *p* = 0.024). Pairwise comparisons indicated that T2 exhibited significantly higher survival probabilities compared to T3 (*p* = 0.026) and T4 (*p* = 0.007). However, no significant difference was observed between T2 and T1 (*p* = 0.251), nor among T1, T3 (*p* = 0.203) and T4 (*p* = 0.076), indicating comparable survival in these groups.

Survival curves demonstrated that T2 (1 g bio‐ox/70 L) achieved the highest CPS (66.7%), followed by T1 (40%), T3 (26.7%) and T4 (13.3%). T4 exhibited the lowest survival, with a steep decline in survival probability, whereas T1 and T3 showed intermediate survival without significant differences. These results highlight the effectiveness of bio‐ox at 1 g/70 L (T2) in improving survival conditions, whereas higher concentrations (T3 and T4) adversely impacted survival.

## 4 Discussion

To the best of our knowledge, this is the first experiment conducted to explore the effects of immediate oxygen supplementation on water quality parameters, behavioural observation and survival percentage of experimental fish in a controlled environment. Bio‐ox, an instant oxygen supplier, has a significant role in maintaining the optimum water quality and the survival of *P. sophore* in the captive condition. However, the identification of appropriate dosage of bio‐ox into water is extremely important and needed to promote water environment and optimize fish production. Thus, the present study was designed to assess the effects of graded levels of bio‐ox on variables related to tank water quality, fish behaviour activity and per cent cumulative survival as well as determine the suitable levels of bio‐ox dosages in the practical field of aquaculture sector.

Suma et al. (2023) documented that the success of aquaculture productivity is heavily dependent on the tank water hydro‐ecological parameters. Ensuring optimal hydrological variables is crucial for enhancing health and production of fish and mitigating disease occurrences in the rearing environment. In this present research, it has shown that different dosages of bio‐ox had substantial impacts on the most of the water quality parameters in terms of conductivity, TDS, total ammonia, DO and pH content. The TDS level was highly significant among the treated groups of bio‐ox over the experimental period and increased the values with the raise of bio‐ox supplementation, indicating an adverse impact on water quality. Bio‐ox is a synthetic chemical that releases chemical compounds or substances when dissolved into water, which is likely accredited to the highest amount of TDS in T4 treatment. TDS consists of minerals and organic compounds that can provide nutrients or harmful substances, including poisonous materials and contaminants, leading to toxicity due to altered water ionic composition and specific ion toxicity (Weber‐Scannell et al. 2007). The presence of high TDS in water can greatly influence the growth and survival rate of fish during their early developmental phase (Mueller et al. 2017).

Correspondingly, water conductivity was significantly enhanced in T4 group as compared to other treatment tanks in this study. Our findings align with previous studies by Abowei (2010) and Ekubo and Abowei (2011), who noted a positive correlation between TDS and conductivity, indicating that higher TDS content leads to increased water conductivity.

The current observation also revealed that total ammonia content was greatly affected by the different bio‐ox dosages and the significantly (*p* < 0.05) higher total ammonia level recorded in the control group. This outcome suggests that various dosages of bio‐ox into water had beneficial effect on water quality by effectively reducing the harmful ammonia load. Nevertheless, excessive ammonia content leads to detrimental impacts on fish, including impaired gill tissue and decreased growth, nutrient efficiency and disease resistance of fish (Hargreaves and Tucker 2004). Fish that suffers from toxic ammonia usually appear lethargic and frequently gasp for air near the water surface (Bhatnagar and Devi 2013).

Gradient supplies of bio‐ox showed considerable fluctuation of DO concentrations. The result of this study indicated that the mean value of DO was notably higher in T4 group among all the bio‐ox treatments. The supplementation of bio‐ox at a rate of 5 g/70 L water exceeds the optimum DO levels, indicating water hyperoxia, whereas the T1 treatment exhibits the lowest DO, demonstrating a state of hypoxia. Hanh et al. (2005) reported that calcium peroxide (CaO_2_), an oxygen‐releasing compound, not only enhances oxygen availability but also stimulates microbial activity. Fish physiological status undergoes significant consequences in response to both hyperoxic and hypoxic states (Bulbul Ali and Mishra 2022). According to Bhatnagar and Singh (2010) and Ekubo and Abowei (2011), DO range of about 5 mg/L is suitable for fish production, which is only found in T2 treatment. However, insufficient DO led to decreased growth and feed intake and increased the mortality of fish (Hargreaves and Tucker 2004). Our findings on bio‐ox as oxygen‐releasing agents in aquaculture align with recent studies in tissue engineering, where materials, like sodium percarbonate (SPO) and calcium peroxide (CPO), are used to supply oxygen under hypoxic conditions (Ke et al. 2022). Both fields highlight that a controlled and sustained oxygen release is crucial—too rapid or excessive oxygen release can lead to cytotoxic effects, as seen with CPO despite catalase treatment. Similarly, in our study, excessive bio‐ox doses caused water hyperoxia and negative impacts on fish behaviour and survival. This parallel underscores the necessity of optimizing oxygen release rates and dosages to balance effective oxygen supplementation with the minimization of adverse effects, whether in engineered tissues or aquatic systems.

Water pH plays a key role in fish farming as it influences the growth, survival, reproduction and overall health condition of fish. The present study found that varied levels of bio‐ox had significant impacts on water pH, and the mean value of it was increased with the rise of supplementation levels of bio‐ox. The observed pH of water in T1 and T2 treatment groups was compared with prescribed limit of pH by Ahmed (2012), Abumourad et al. (2013) and Gan et al. (2013). However, elevated pH had detrimental effects on growth and reproductive performance of fish (Zweig et al. 1999) and resulted in substantial fish mortality (Zahangir et al. 2015). In a study, Sahu and Datta (2018) found that pH levels below 5.71 and above 8.20 caused acute stress and resulted in a 50% fish mortality.

In this investigation, the other hydro‐ecological parameters such as temperature, water pressure and salinity revealed no noteworthy variations among the treatment groups. This suggests that oxygen supplement had no potential influences on these particular parameters. However, the results found in this study align with the optimal levels for fish production as reported by numerous researchers (Santhos and Singh 2007; Kabir et al. 2024; Rahman et al. 2023). Although numerous studies (Dutta 1996; Clotfelter and Rodriguez 2006; Pandey et al. 2011; Selvi and Ilavazhahan 2015; Renick et al. 2016; Stalin et al. 2019; Moreira et al. 2021) are extensively documented the impacts of various levels of synthetic chemical substances like pesticides, insecticides or herbicides on fish behaviour, the full understanding of the effects of bio‐ox on it is still lacking. Observing fish behaviour in aquaculture is vital for health monitoring, assessing environmental conditions, optimizing feeding efficiency, detecting early signs of issues and ensuring successful fish culture management. The current research identified a deviation in the swimming patterns of *P. sophore* during a 72‐h observations period when subjected to different concentrations of bio‐ox dosages. Fish in the T4 treatment group displayed significant changes in behaviour, including hyperactivity, abnormal swimming, loss of equilibrium, mucus secretion, operculum movement, reduced feeding rate, depigmentation and convulsions. In contrast, fish treated with T2 treatment exhibited normal behaviour. The overall findings suggested that fish behaviour is predominantly influenced by optimal bio‐ox levels in water.

**FIGURE 3:**
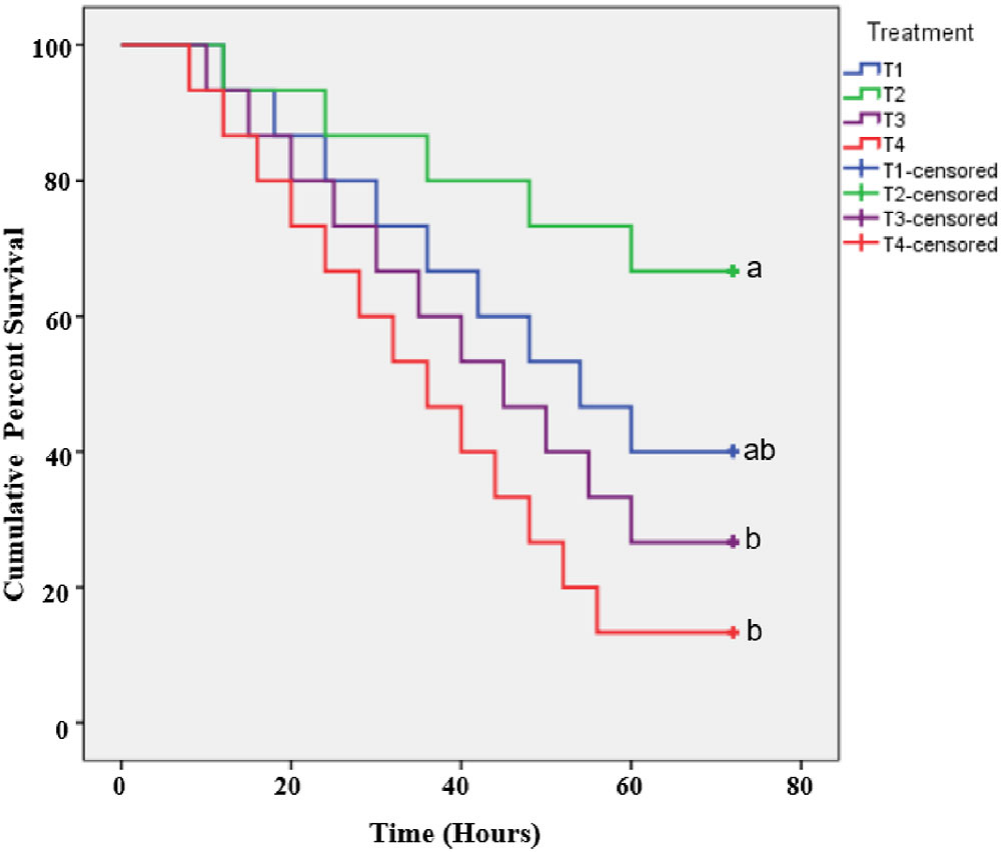
Kaplan–Meier survival curves of *Puntius sophore* exposed to various dosages of bio‐ox. Survival is represented as cumulative per cent survival over the 72‐h experimental period for the four treatment groups (T1, T2, T3 and T4). Censored cases are marked with “+” symbols, and significant differences were observed among the treatments (*p* < 0.05).

The Kaplan–Meier survival analysis revealed significant differences in CPS among the bio‐ox treatment groups (Figure 3). The T2 group demonstrated the highest survival percentage (66.7%), significantly exceeding those of T3 (26.7%) and T4 (13.3%). These results underscore the beneficial effect of a low bio‐ox dosage (1 g/70 L) in improving fish survival. In contrast, the T4 group exhibited the lowest survival, indicating that excessive bio‐ox dosages adversely affect water quality and fish health. T1 (control) showed intermediate survival but was outperformed by T2, suggesting that even minimal oxygen supplementation can yield positive results under controlled aquaculture conditions.

Distinct differences in the survival rates of *P. sophore* were observed across the bio‐ox treatment groups, as evidenced by the Kaplan–Meier survival analysis (Figure 3). The lowest survival percentage was observed in the T4 group, whereas the T2 group exhibited the highest survival, highlighting the benefits of a low dosage of bio‐ox in improving fish survival. In contrast, higher dosages of bio‐ox (T3 and T4) adversely affected water quality, particularly DO and pH levels, potentially leading to higher mortality. Although DO levels remained higher in the T4 group due to the higher dose of bio‐ox, it also caused a noticeable rise in pH levels. As seen in Figure 2, the pH levels in T4 remained elevated from 12 to 66 h. Although higher DO levels are generally beneficial, excessive levels can disrupt water chemistry and significantly alter pH, potentially creating unfavourable conditions for fish survival. Sheikh and Slathia (2018) documented that the lowest DO result in sudden mortality of major carps (*Cyprinus carpio communis* and *C. carpio specularis*). Similar effects were also viewed in Central Kenya when fish were subjected to high content of ammonia in water (Wanja et al. 2020). Thus, the appropriate dosage of bio‐ox is critical for maintaining optimal water quality and ensuring the survival of *P. sophore*, highlighting the importance of balanced oxygen supplementation in aquaculture practices.

## 5 Conclusions

On the basis of the current findings, it can be suggested that the supplementation of bio‐ox at approximately 1 g/70 L of water (T2) improved the tank water quality parameters, particularly in terms of DO and pH. This supplementation also positively affected fish behaviour, leading to a decrease in mortality rates in captivity. Moreover, the findings from this study could provide valuable insights for emergency oxygenation strategies in regions with unreliable electricity and aid farmers in determining the optimal dosage and application of bio‐ox in water. However, further trials are needed to find the best dosage and application method and understand the long‐term effects of bio‐ox supplementation. Moreover, its impacts on fish growth, feed use, health and cost‐effectiveness should be studied to fully understand the benefits and challenges of using oxygen supplementation in large‐scale aquaculture practices.

## Author Contributions

Md. Abdullah Al Mamun led the conceptualization, validation, project administration, statistical analysis and contributed to funding acquisition and writing the original draft. Shishir Kumar Nandi handled the methodology, software, formal analysis and contributed to writing the original draft. Arman Hossain contributed to the methodology, data curation and investigation. Shamima Nasren supervised the project and contributed to funding acquisition, writing review and editing. Muhammad Anamul Kabir contributed to visualization and writing review and editing.

## Ethics Statement

The Animal Ethics Committee of Sylhet Agricultural University provided guidelines for the handling of fish in this study and use of antibiotics (Memo: SAU/AEC/FOF/FHM‐569), which were strictly followed.

## Conflicts of Interest

The authors declare no conflicts of interest.

## Peer Review

The peer review history for this article is available at https://publons.com/publon/10.1002/vms3.70624.

## Data Availability

Data are available from the corresponding author on request.
